# *In vitro* Models of the Blood–Brain Barrier: Tools in Translational Medicine

**DOI:** 10.3389/fmedt.2020.623950

**Published:** 2021-02-15

**Authors:** Alberto Williams-Medina, Michael Deblock, Damir Janigro

**Affiliations:** ^1^Department of Physiology and Biophysics, Case Western Reserve University, Cleveland, OH, United States; ^2^Flocel, Inc., Cleveland, OH, United States; ^3^Department of Biomedical Engineering, Cleveland Clinic Foundation, Cleveland, OH, United States

**Keywords:** *in vitro*, translational medicine, CNS drug delivery, Alzheimer's disease, COVID-19, microvesicles, multiple sclerosis, graphene

## Abstract

Medical progress has historically depended on scientific discoveries. Until recently, science was driven by technological advancements that, once translated to the clinic, fostered new treatments and interventions. More recently, technology-driven medical progress has often outpaced laboratory research. For example, intravascular devices, pacemakers for the heart and brain, spinal cord stimulators, and surgical robots are used routinely to treat a variety of diseases. The rapid expansion of science into ever more advanced molecular and genetic mechanisms of disease has often distanced laboratory-based research from day-to-day clinical realities that remain based on evidence and outcomes. A recognized reason for this hiatus is the lack of laboratory tools that recapitulate the clinical reality faced by physicians and surgeons. To overcome this, the NIH and FDA have in the recent past joined forces to support the development of a “human-on-a-chip” that will allow research scientists to perform experiments on a realistic replica when testing the effectiveness of novel experimental therapies. The development of a “human-on-a-chip” rests on the capacity to grow *in vitro* various organs-on-a-chip, connected with appropriate vascular supplies and nerves, and our ability to measure and perform experiments on these virtually invisible organs. One of the tissue structures to be scaled down on a chip is the human blood–brain barrier. This review gives a historical perspective on *in vitro* models of the BBB and summarizes the most recent 3D models that attempt to fill the gap between research modeling and patient care. We also present a summary of how these *in vitro* models of the BBB can be applied to study human brain diseases and their treatments. We have chosen NeuroAIDS, COVID-19, multiple sclerosis, and Alzheimer's disease as examples of *in vitro* model application to neurological disorders. Major insight pertaining to these illnesses as a consequence of more profound understanding of the BBB can reveal new avenues for the development of diagnostics, more efficient therapies, and definitive clarity of disease etiology and pathological progression.

## Introduction

The human blood–brain barrier (BBB) is an exceedingly important histological barrier that controls the interplay, communication, and molecular trafficking between the CNS and the periphery. The cells that compose the BBB (astrocytes, vascular endothelial cells, and pericytes) bring about the neurovascular unit and are therefore directly (and indirectly) involved in the regulation of cerebral blood flow (CBF) and, by consequence, neuronal activity (1). The cellular and molecular features of the BBB have been reviewed elsewhere (2–7) and are beyond the scope of this review.

Repercussions of BBB disruption result in manifestations that are most noticeable *in vivo*. Many of these repercussions are described as signs and symptoms of disease in the clinical setting, of which mostly are modeled in rodent research. However, *in vivo* modeling mainly sheds light onto the behavioral and systemic effects of, in this case, BBB disruption. *In vitro* models provide an excellent framework to define and identify key cellular/molecular players, targets, and regulators that complement findings obtained *in vivo*. In addition, it has become more necessary to develop models *in vitro* that can render data often required to be performed *in vivo*. Thus, innovative modeling of the BBB *in vitro* can facilitate the comprehensive study of its fluidity, regulation, and integrity while bridging the gap between strictly *in vivo* and *in vitro* findings. Elucidating the degree to which the etiology and pathology of neurological illness is due to BBB disruption not only is of extreme relevance but also is a great opportunity for the development of directed therapies, disease prevention, and improvement of medical practices. To further extend the reach of research in this field, the focus needs not to be exclusively narrowed to basic science of disease or model design, but rather incorporate both means onto the same end. In other words, BBB research should encompass defining the mechanisms of BBB disturbance in concert to devising (and comparing) *in vitro* models that more closely resemble the BBB environment (and vice versa). Our article seeks to recapitulate the progress of BBB research highlighting the development of *in vitro* models up to the present and suggesting next steps in model design to mimic the environment of the BBB more closely. Major advances in neurodegenerative disease modeling are also discussed [multiple sclerosis (MS) and Alzheimer's disease (AD)] as well as the involvement of infectious disease [coronavirus disease 2019 (COVID-19)] in neurological illness.

### Recently Established *in vitro* Models of the BBB

Modeling the BBB *in vitro* has become more necessary for the advancement of neuroscience research. Despite *in vivo* studies yielding insight into the systemic effects brought by BBB disruption, it is not a tool to evaluate the cellular/molecular interplay between the cells that compose the BBB and the neurovascular unit. In order to elucidate the mechanistic properties that govern the maintenance and genesis of the BBB, more technologies must be designed to model its physiologic environment more closely. Transwell studies are widely used and have provided a great array of knowledge about the dynamics of the BBB; however, its static nature limits the extent to which results can accurately model its physiology. It is for this reason that dynamic models of the BBB are absolutely crucial toward lengthening strides in research to further define the fluid properties of the BBB. Here, we outline current *in vitro* models of the BBB that have advanced the field and pose as good alternatives to the Transwell model.

#### Organ-on-a-Chip (OACC)

Organ-on-a-chip (OOAC) technology has become more widely used in recent years due to its ease of use and ability to mimic physiological conditions (often performed *in vivo*) in an *in vitro* setting (8–12). The model consists of a 3D layered system (similar to a Transwell ensemble) on an enclosed microscale formfactor (chip) and employing flow of cell media (designs may vary) (13). These ensembles may also include histological matrices (i.e., extracellular matrix complexes, basement membrane elements, etc.) to better resemble the physiological environment of tissues/organs. This allows the recreation of tissue barriers *in vitro* with the added feature of establishing microfluidic circuits to perfuse modeled organs across an array of multiple, interconnected chips, thus better modeling the physiological crosstalk and circulation across organ systems. Moreover, OOACs can be cultured with human cell lines, allowing researchers to more closely model human organ/tissue systems without the need to recur to animal cell lines. This is particularly well for studying the BBB as it allows researchers to replicate the histology of the structure as OOAC setups using primary cell culture lines or inducible pluripotent stem cells (iPSCs) (12, 14). Current research employing the use of OOACs mainly revolve around targeted drug discovery and delivery with few examples in the basic science literature regarding the functionality of the BBB and its implications in human neurological illness. References (13, 14) describe the development and design of OOAC models more extensively. We emphasize the use of OOACs as a potential *in vitro* model for studying the BBB as it can complement and advance the study of the brain and neurological disease beyond solely experimental model design and treatment development. Albeit extremely important, basic neuroscience knowledge is especially needed at the bedside, primarily for improving diagnostics and extending preventative medicine. Moreover, multiple clinical scenarios of neuropathology could be explored utilizing OOAC models to shed light onto the ill-defined etiology of neurodegenerative disease (refer to the section *Translation to Human Disease*).

#### Organoids

Studying organ and tissue systems *in vitro* has been a major hurdle for neuroscience research. Many projects that seek to further define neurological illness often have to rely on animal models to be able to replicate and observe phenomena at the organ/tissue level. This involves tedious and long-term rodent work. *In vivo* protocols being subject to institutional and federal review boards, along with facility costs, add more considerations to the scope of the intended study. In past decades, *in vitro* model designs of organs and tissues derived from stem cells in 3D ensembles called *organoids* have shown much promise for advancing neuroscience research and outpacing *in vivo* models. The main idea consists of differentiating pluripotent stem cells (PSCs) or adult stem cells (AdSCs) into embryonic tissue layers (i.e., ectoderm, mesoderm, and endoderm) and presenting the stem cell culture with factors that will further direct their maturation into the tissue/organ of choice (of which provide great cellular diversity) (15, 16). More detailed discussion over the history, design, and methods for making organoids are covered elsewhere (15–18). Much more attention has been drawn to human cell-derived organoids. Emergent BBB organoids should also be considered in coming research as these can provide much data comparable to *in vivo* studies. The complexity of the neurovascular unit and its physiological constraints could be investigated more closely employing the use of organoid *in vitro* models, with special interest on how the BBB is responding to neurological pathology and drug delivery. Further review regarding organ-specific examples of organoid models for translational research can be accessed in reference (17).

### A Brief History of *in vitro* Models of the BBB

**Figure 1:**
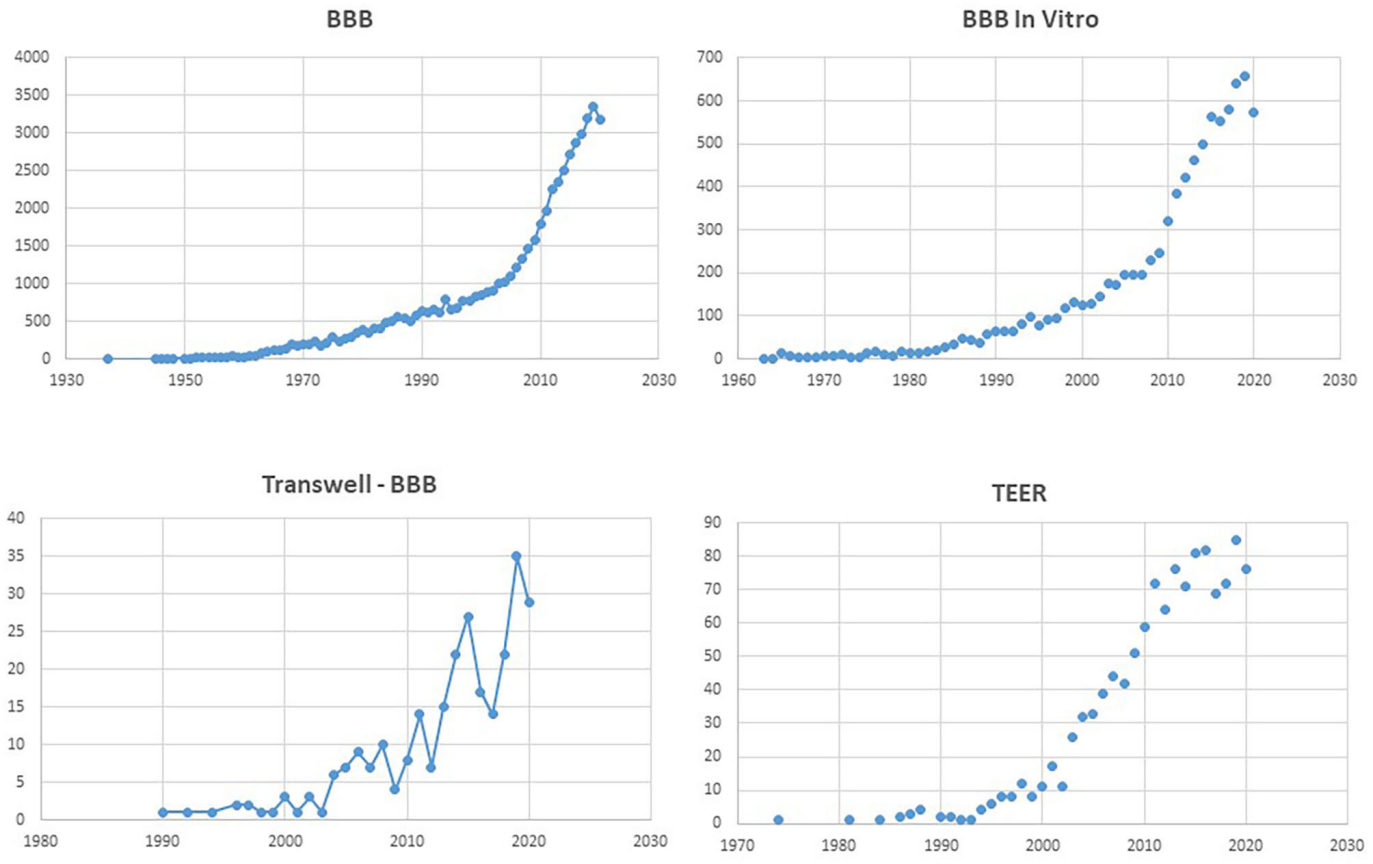
Number of PubMed hits for the terms listed as title. Note the exponential increase for “BBB,” and the comparable profile for “BBB *in vitro*.” Remarkably, the search for “Transwell BBB” and “TEER” has a similar temporal increase as “BBB *in vitro*,” underscoring the popularity of the Transwell apparatus.

**Figure 2:**
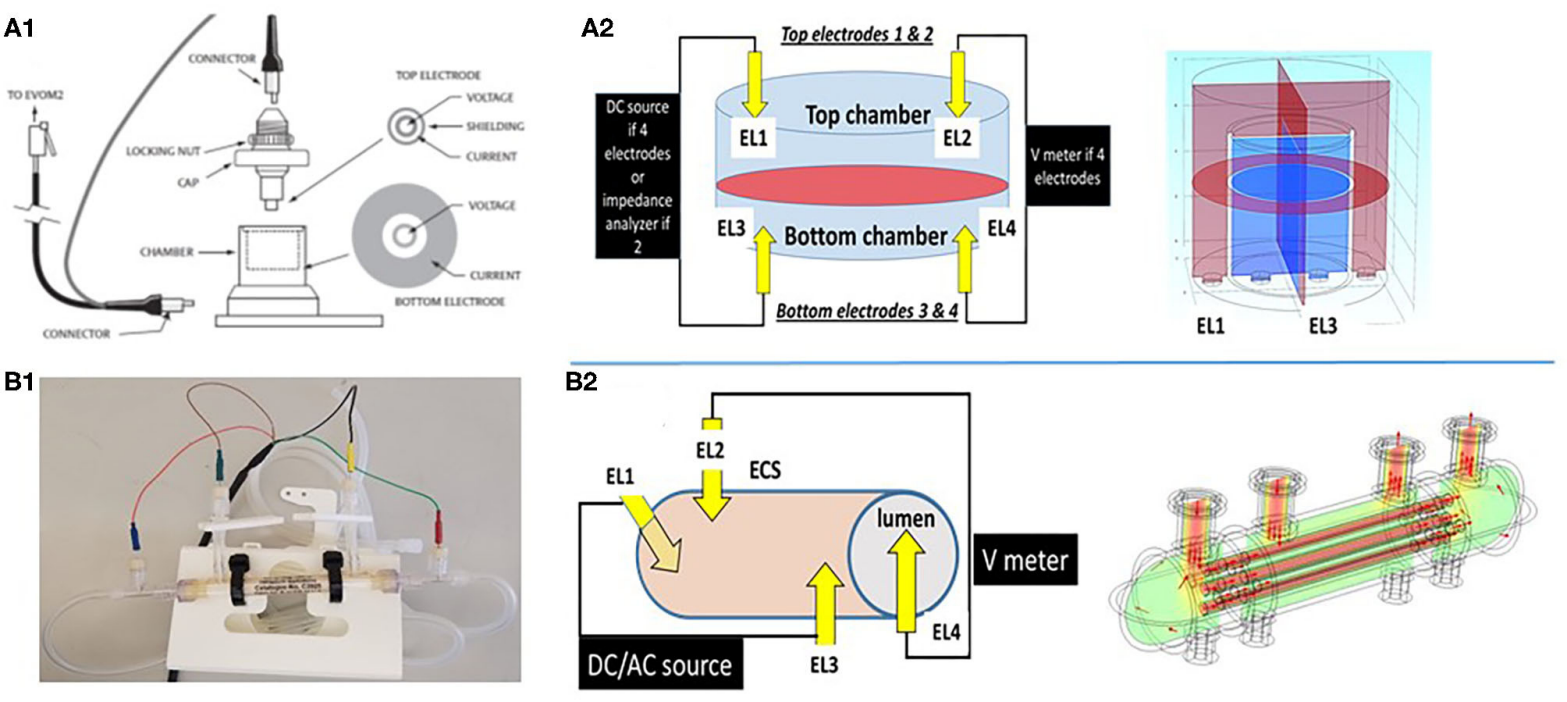
Schematic depiction of two different configurations used to grow cerebrovascular cells to mimic the blood–brain barrier. **(A1,A2)** shows a Transwell device and the settings used to measure TEER. Owing to its geometry and the placement of electrodes close to the monolayers, the control of voltage across the cell monolayer is uniform. This was modeled by the multiphysics model COMSOL (Version 4.3b) as shown in the upper right panel. In the 3D configuration **(B1,B2)**, the control of voltage is also continuous owing to the four-electrode configuration used. Thus, two or four electrode systems have been used to measure impedance *in vitro*. We measured trans-endothelial electrical resistance under many different conditions and with several different electronic devices. However, when modeling the behavior of the two electrode configurations, we found that while this approach is reliable for 2D models, it fails to satisfactorily control voltage on a more sophisticated 3D model (Figure 3). Note that for the 2D model, the electrodes had a surface area comparable to the area of the layer across which resistance was measured, while for the 3D model imitating a blood vessel, the electrodes consisted of small point contacts. These are the contacts used for organs-on-a-chip, since complex geometries disallow coating the structure with large closely spaced electrode contacts. We propose to use a four-point, AC system (as in the DIV-BBB; see also Figure 3) to overcome this.

The most common *in vitro* model of the BBB is based on the Transwell apparatus (Figures 1, 2) (19, 20). The first reported use of endothelial cells on a transparent collagen filter was developed by a few groups in the early 1990s (21, 22). In the following years, the Transwell technology remained essentially the same, although materials employed differed substantially. The inset filters used have variable porosity and composition, allowing for cell extravasation, if so desired. Initially, Transwell apparatuses were used for monocultures of either endothelial or epithelial cells (e.g., Caco-2, MDCK), but the configuration of the Transwell allows for different modalities of co-culture. For example, endothelial cells are typically grown on the top of the filter, while secondary cultures are seeded on the opposite side of the filter (contact co-culture) or at the bottom of the well (non-contact co-culture) (20, 23–25). In addition, conditioned media have been used as differentiating factors, whereby media from a traditional petri dish culture are used to influence endothelial cell differentiation in the Transwell (26–28). The popularity of this approach for *in vitro* modeling is due to its ease of use, availability in multi-inserts for high-throughput screening, and the fact that cells are visible to the operator during the process and after fixation and staining *in situ*. There are several recognized drawbacks (see below), but the longevity of this simple approach remains virtually unchallenged (Figure 1).

Monitoring the development and integrity of cell barriers during maturation and experiments is crucial for all studies performed on barriers. Methods for assessing barrier permeability are based on the transport of tracer substances (e.g., mannose, sucrose, dextrans, or fluorescent dyes bound to protein, immunofluorescent staining of proteins related to the tight junction complex) or measurement of trans-endothelial electrical resistance (TEER) (20, 25, 29–34). Electrical resistance rises from the high transport resistance of ions through a cell layer and can, therefore, be used as a measure of the integrity of a barrier. In a way, the electrical resistance of a cell under whole cell patch clamp conditions is analogous to TEER inasmuch it is due to the low permeability of lipid layers to electrical current flow, which increases when membrane “leakage” is present: the difference in BBB studies is that TEER refers to a determination of a “leak” pathway between cells (the paracellular leak pathway) usually “blocked” by tight junctions. It has to be noted that, by design, the Transwell approach provides a large “paracellular leak” at the edge of the monolayer, since tight junctions do not form between cells and plastic (24). This is one of the reasons behind some of the non-physiological aspects of BBB models grown in these inserts (high permeability to sucrose, low TEER). A major engine behind Transwell's success is the availability of a simplified solution to measure TEER (or epithelial monolayer resistance; see Figures 1, 2). The biological background of TEER measurements was reported in the 1980s (35). Alternating- and direct-current electrical characteristics of rabbit corneal endothelia were studied under varying experimental conditions. The approach used to measure resistance (or, more correctly, impedance) is similar to the more modern setup used for Transwell/Endohm/Evohm (World Precision Instruments, Sarasota, FL; Figure 2) and consistent with methods employed *in vivo* (36–39). In brief, resistance is measured by applying to one side of the e-monolayer an electrical signal (*I*, current or *V*, voltage) and by measuring the corresponding voltage (or current) on the other side. Using Ohm's law (*V* = *I*/*R*, where *R* is the resistance), the measurement is easy to perform and no complex mathematical equations need to be solved. The astute “plunger” configuration of the Endohm (Figure 2A1) fits several dimensions of Transwell inserts and the operator's involvement in the measurement of TEER consists of switching the measurement on and off and taking a manual note of the value recorded.

Despite its ease of use and popularity, the Transwell/Endohm system has several shortcomings. For example, the cells grown on filters are not exposed to “blood” flow and therefore lack the influence of shear stress on endothelial cell differentiation (20, 30–32, 40–42). In addition, the 2D morphology of a Transwell does not recapitulate the anatomy of cerebral vessels or brain capillaries. The volumetric relationship between “brain” and “vascular” side volumes is also a poor reproduction of what is seen *in vivo* [e.g., 6 L of blood and 150 ml of cerebrospinal fluid (CSF), vs. a roughly 1:2 relationship, brain having more volume than blood side], which is a confounder for drug permeation studies. Last, the TEER values measured with Endohm/Evohm are significantly lower than those measured *in vivo* or in other systems (20, 24, 30) possibly due to the lack of the differentiating influence of flow exposure [see, for example, (43); see also above]. In addition, the electronic circuit used to drive TEER measurements with Transwell-Endohm apparatuses suffers from a major flaw, i.e., resistance (or more accurately, impedance) is measured only at a single frequency and at prefixed “up-down” DC steps. The relevance of multifrequency scans and use of AC instead of DC for the determination of transcellular impedance and the limits of TEER applied to Transwell are described in detail elsewhere (25, 29, 44, 45).

The dynamic *in vitro* model of the BBB (DIV-BBB) has been compared to the Transwell system, to demonstrate a number of advantages including a higher TEER value (20), a pulsatile physiological release of nitric oxide (46, 47), a different profile in gene expression (31, 40, 48), as well as a more realistic profile of drug permeation (20, 33, 49–53). The dynamic nature of the DIV-BBB apparatus also allowed the study of the effects of normoxic/hypoxic flow cessations and reperfusion (32), as well as the effects of circulating leukocytes on BBB integrity under flow/no-flow reperfusion conditions (54). In addition, the effects of viral infection (simian immunodeficiency virus) was investigated using a long-term (several months) culture of endothelial cells and glia (55–57). A significant difference in cell viability, survival, and metabolic activity in the DIV-BBB recapitulates the *in vivo* conditions more accurately than the Transwell. This is in particularly true for the metabolic use of oxygen and glucose, which is mostly anaerobic in Transwell but 50% aerobic in the DIV-BBB [e.g., (20)]. This was shown by measuring the conversion of glucose to lactate under steady-state growth conditions. Thus, cells grown in the presence of an unstirred layer (Transwell) adapt to chronic hypoxic conditions, while endothelial cells *in vivo* are exposed to blood pO_2_ levels, which are continuously kept at physiological levels by gas permeant tubing and fluid mobility. Lastly, the DIV-BBB has been successfully used to compare the profiles of mechanisms of multiple drug resistance (MDR) in human epileptic brain vs. a humanized DIV-BBB (49, 50, 52, 58–63) (for a comparison with a rodent study, see Figure 6).

**Figure 3:**
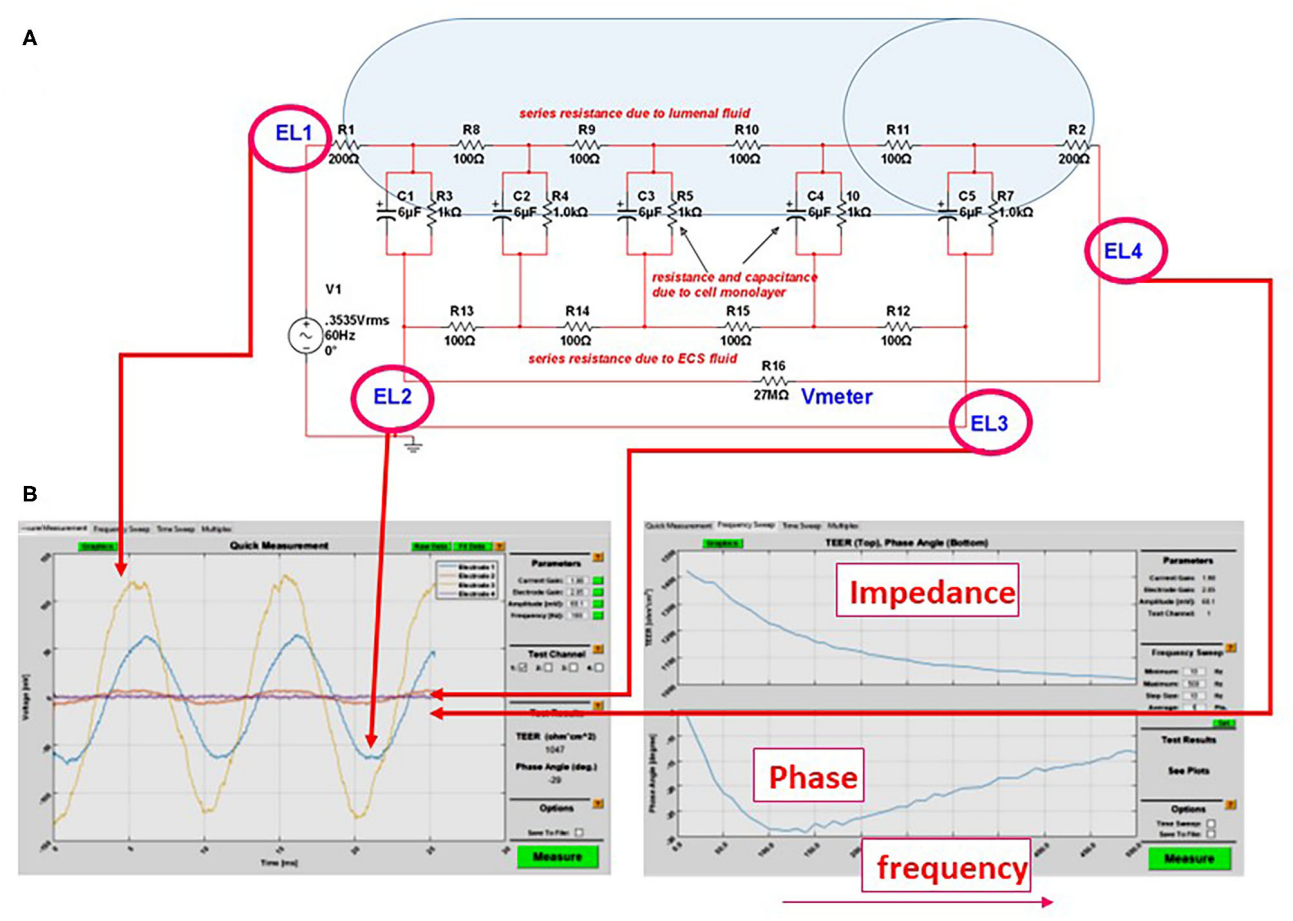
**(A)** Equivalent circuit we used to predict the accuracy or measurements of transcellular resistance in different 3D organs-on-a-chip. For example, the shaded area in the figure represents the size of a venule (~200 μm diameter). In this model and with an AC, four-electrode clamp approach, the resistance measured is accurate. This is achieved by measuring the relative contribution of various resistive components and by subtracting background resistance from the total to obtain cell TEER. **(B)** Output of the DIV-BBB TEER software based with Matlab. Note the four waveforms measured at the electrodes EL1–EL4 in **(A)** and the impedance/phase diagram across a broad range of frequencies.

**Figure 4:**
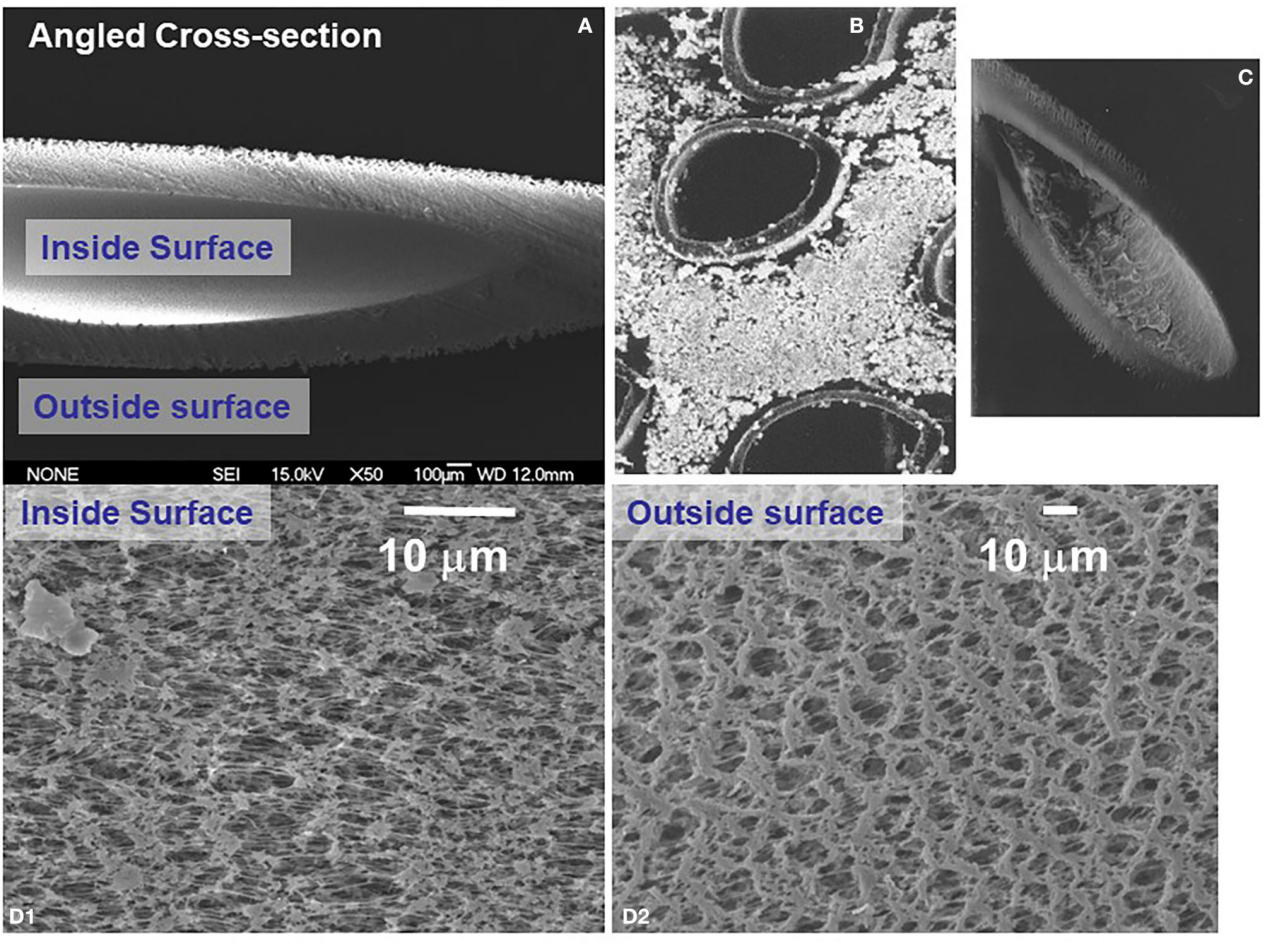
Scanning electron microscope appearance of hollow fibers used to dynamically grow endothelial and astrocytic co-cultures. **(A)** Shows a transverse section of an empty fiber, while **(B,C)** shows a number of fibers growing abluminal astrocytes and (inset) endothelial cells. **(D)** Shows the porosity of the outer (D2) and inner (D1) surface of a typical polypropylene hollow fiber used for the DIV-BBB apparatus.

**Figure 5:**
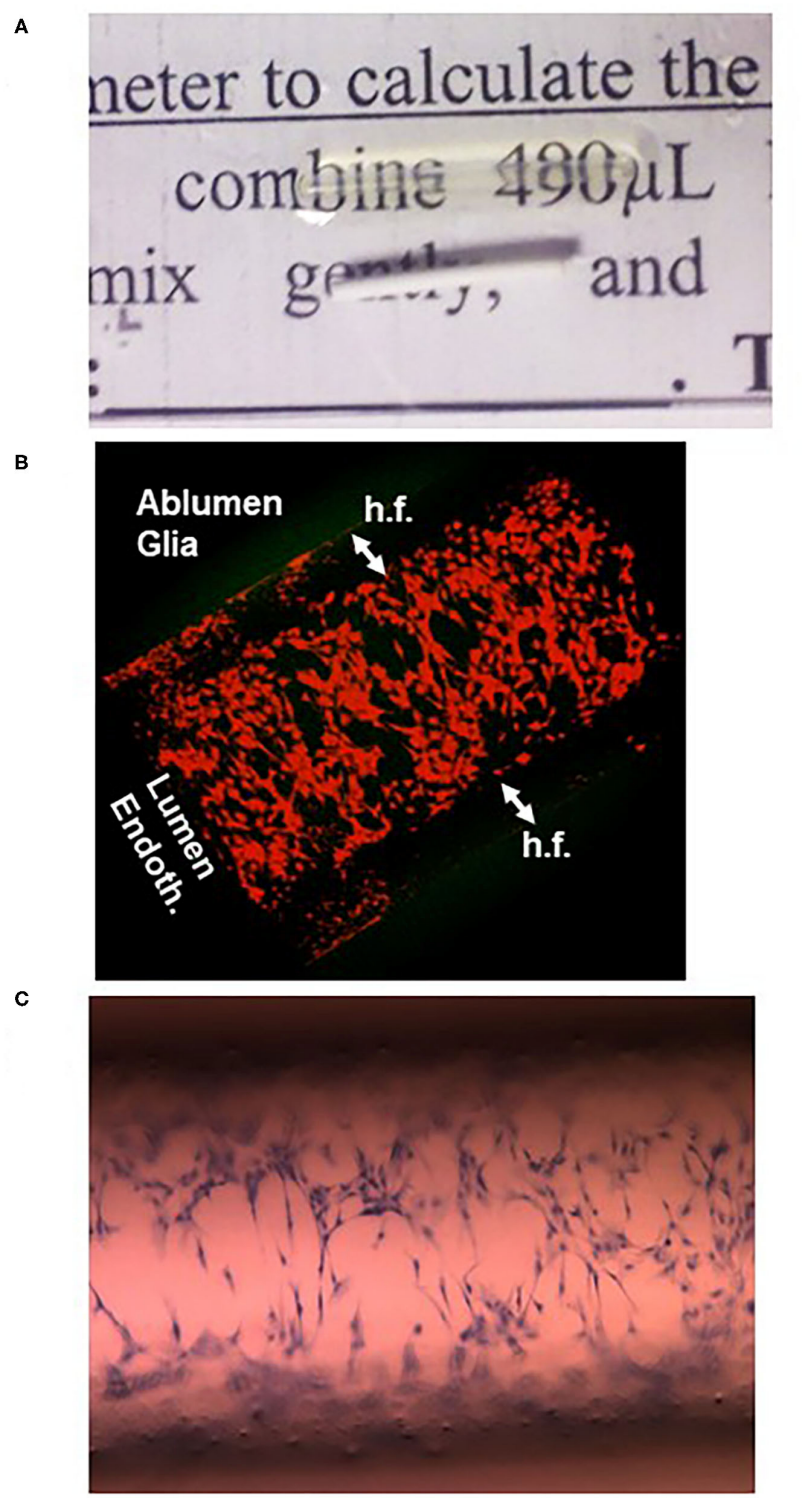
One of the main limitations of the hollow fiber is their optical property that does not allow intralumenal inspection during an experiment. However, after immersion for a few minutes in mineral oil used for microscopy, the fibers become transparent **(A)** and can be visualized by confocal **(B)** or light **(C)** microscopy. The cells in **(B)** are endothelial in the lumen of the hollow fiber (h.f.) and astrocytes in the ablumen; both were colored with a red cell tracking dye. In **(C)**, cresyl violet staining of sparse endothelial cells 1 h after plating is shown.

**Figure 6:**
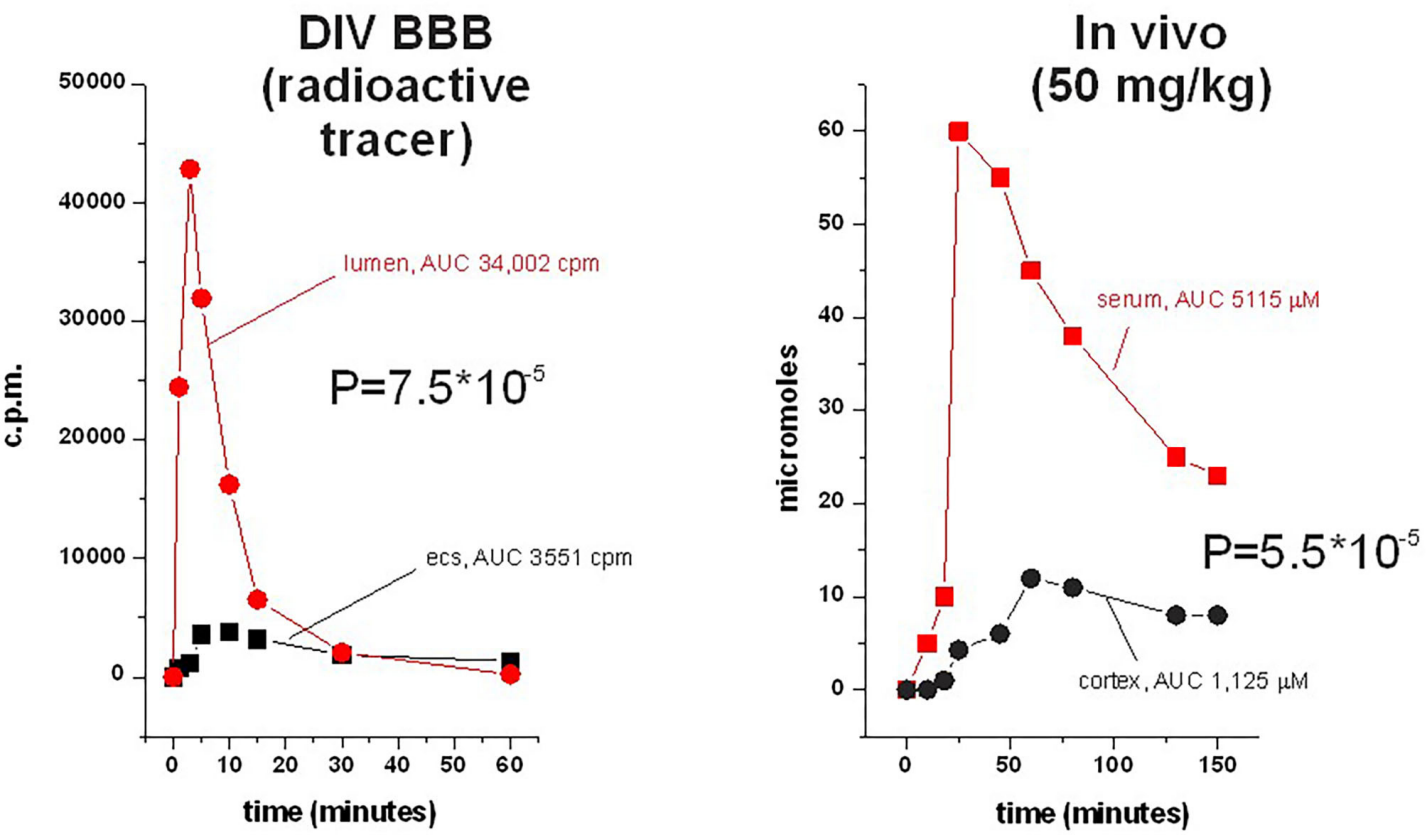
Side-by-side comparison of permeation of phenytoin across the DIB-BBB or in the rat brain. Brain penetration was calculated as a permeability as described, and 3H-phenytoin and cold phenytoin were used for *in vitro* and *in vivo*, respectively. The levels in brain and blood were measured by microanalysis; similar sampling was used in the DIV-BBB. Note the similar permeation profiles in the dynamic model compared to the rodent.

The first attempt to culture endothelial cells under dynamic (flow) conditions with a quasi-physiologic shear stress and cells grown with a vascular geometry in mind was achieved by Ballerman et al. (64, 65) using hollow fiber technology. This approach was further developed by us in the 90s (66–69) and more recently by us (20, 32, 33, 48, 52, 63, 70–72) and others (46, 47, 73–76). The so-called dynamic *in vitro* model of the BBB (DIV-BBB; Figures 2–6) consists of several hollow fibers packaged in plastic modules. The microscopic appearance of the hollow fibers is shown in Figure 4. Using the fibers shown in the figure, cell extravasation is impeded (cutoff around 0.5 μm); however, larger porosities allowing for leukocyte extravasation can be obtained with a variety of methods [e.g., (30, 77); see graphene hollow fibers under section *Conclusions and Future Directions*].

Since the two-electrode setup of Evohm is not appropriate for measurements in a 3D culture system because of poor *V*- or *I*-clamp properties [see References (24, 78, 79); a four-electrode impedance TEER apparatus developed by one of us (DJ) has been used since 1996 [e.g., (66)]. The control of voltage profile and the ease of recording both phase and impedance (Figures 2, 3) have made this system an integral part of the DIV-BBB models of the BBB. Several variations and improvements have been developed during the past 20 years, but the four-electrode approach is still used for 3D models with a vascular geometry or a capillary–venous configuration (20, 24, 30, 31, 71, 77–80).

As with every model, limitations of the DIV-BBB approach were also noted. First, establishment and maintenance of the DIV-BBB are complex and more labor-intensive than use of Transwell technology. In addition, the support for cellular growth (usually polypropylene) that constitutes the hollow fibers is not comparable to the biological basal lamina that separates endothelial cells from glia (Figure 4). In addition, the thickness of the wall separating endothelial cells from brain cells (e.g., astrocytes) is exaggerated (>100 μm), albeit permissive for astrocyte end-feet contact with endothelial cells (75). Other limitations, in particular from the perspective of a “brain-on-a-chip” development, is the bulk of the DIV-BBB modules. This has been partially addressed by 3D printing of small, single-fiber modules. The advantage of bulk, however, is the maintenance of pressure and shear levels that reproduce not only the capillary segments of the cerebral vasculature but also pre- and post-capillary segments (30). Lastly, the hollow fiber technology did not allow us to visualize luminal cells. This has been in part alleviated by use of immersion of the fiber in microscopy mineral oil (Figure 5).

### Static vs. Dynamic Models of the BBB

Currently, the Transwell model is the most used *in vitro* BBB model due to its simplicity, ease of use, and straightforward procedures. However, because of its simplicity, these models are unable to accurately replicate physiological environments. Dynamic models of the BBB can better address the complexity of organs/tissues but are more involving with respect to costs and personnel training. More technologies emerge throughout the years that seek to balance the pros and cons of each model or attempt to improve upon the established models to address their limitations. In this section, we will cover the differences and similarities, as well as compare and contrast current static and dynamic models of the BBB while emphasizing possible next steps to direct the field onward.

#### Beyond the DIV-BBB Model

The trend of post-DIV-BBB modeling was aimed at addressing the problems of the dynamic 3D model while also allowing for a modular “insertion” in a human-on-a-chip platform. This implies use of microfluidic methods, also aided by the post-DIV-BBB widespread availability of 3D printing [including two-photon lithography (81)]. One of the earliest microfluidic attempts (24) directly addressed one of the limitations of the DIV-BBB, namely, the large distance between endothelial cells and perivascular cellular elements. A pitfall of the system was the use of a transformed endothelioma cell line in lieu of brain (or peripheral) endothelial cells. Notwithstanding, the results of a comparison with a Transwell culture were encouraging, and the authors were able to repurpose the Transwell TEER measurement system to measure TEER with a dual electrode plate configuration that was appropriate for the geometry of their planar system. The “vasculature” was however failing the native cylinder geometry of blood vessels, as in most of future microfluidic attempts. This creates an edge between materials used to shape the vascular cavity and the cells, in a way that resembles what is seen in a Transwell. When the whole vascular cavity (shaped as a parallelepiped) is covered by cells, this problem is avoided, but exposure to shearing forces, if any, becomes difficult to compare and is somewhat dishomogeneous. Several variants on the microfluidic BBB modeling theme are available, including the commercial system Synvivo (https://www.synvivobio.com/). In general, these models allow for excellent visualization of cells during the experiments, the use of multiple cell types including neurons, and the perfusion of various compartments with the physiological fluids required for cellular survival.

A new miniaturized version of the DIV-BBB was recently developed (43, 82) where cells are grown into plastic inserts and exposed to flow. The TEER values of native cells were comparable to those seen in a Transwell, but addition of barrier-inducing media led to a significant improvement of barrier function. In this model, however, shear stress values were at the low end of capillary perfusion, and the images of endothelial cells showed several mitotic cells, which is not expected given that shear stress abolishes cell division (31, 40).

In 2012, the first BBB models derived from human-induced pluripotent stem cells (hiPSCs) were invented and are now reaching a level of validation that might make them suitable for utilization in preclinical drug development programs in the pharmaceutical industry. In addition, these models could provide insights into mechanisms of CNS diseases, which are often associated with general or specific pathophysiological alterations at the BBB [see for review (83)].

A number of novel TEER systems have been paired to these microfluidic models, spanning from the use of Endohm/Evohm adaptations (24, 25) to more sophisticated approaches (84). It has to be noted that in addition to TEER, a barrier viability method that has been frequently employed uses as a parameter permeability to impermeant molecules (typically sucrose or dextrans with high molecular weight). The two approaches are conceptually similar, since they both measure passage of matter across the cell monolayer, but distinct in their use. TEER can be measured at second intervals unobtrusively and non-invasively for days (e.g., the DIV-BBB apparatus), while a tracer permeability study is typically performed at a single type point of an experiment owing to its invasiveness and need for repeated sampling of luminal and brain side fluids.

#### BBB Models and Drug Delivery

Apart from instances in which drugs are introduced directly into the CNS, the concentration of the agent in the blood after oral or parenteral administration differs substantially from its concentration in the brain. Thus, one of the main thrusts behind the push for the development of *in vitro* BBB models has been to produce highly predictable devices and cell aggregates to predict drug passage across the BBB. There are several reviews that discuss the utilization of *in vitro* models of the BBB to study the pharmacokinetic properties of peripherally administered drugs (85–87). We wish to only summarize a few concepts that derive from experience with *in vitro* models of cell-based BBB surrogates. Limiting the discussion to small molecules, we have learned several lessons, including:

1. In general, making small molecules more lipophilic facilitates passage across the BBB. Success stories are the acute use of anxiolytics (e.g., benzodiazepines), serotonin-specific reuptake inhibitors (e.g., Prozac), narcotic pain relievers and opiate derivatives (e.g., oxycodone), drugs of abuse (e.g., heroin), and general anesthetics (i.e., propofol) (88, 89). Mixed results came from the field of anti-epileptic drugs where a large percentage of patients develops multiple drug resistance (63, 90–94).
2. The chemical–physical properties of a drug made lipophilic often allow its extrusion by multiple drug resistance transporters.
3. In addition to anti-epileptic drugs, several drugs are substrates of multiple drug resistance transporters [e.g., chemotherapic/antineoplastic compounds (94)].
4. In most diseases of the CNS, the BBB is compromised: thus, prediction of passage (or not) across a normal BBB is not always useful (89). Perivascular edema and other physicochemical changes in the brain hamper drug distribution particularly in the lesioned brain (temporal lobe epilepsy, neoplasm) (89).
5. The involvement of nanomedicine in BBB experimentation is becoming more apparent and promising for developing targeted diagnostics and therapeutics. Detailed discussion of these applications can be found in the *Translation to Human Disease* section.

Given these factors, how useful are *in vitro* models? Our experience with Transwell and DIV-BBB vs. *in vivo* data have shown that:

1. In general, humanized *in vitro* models are superior to rodent models (33, 71).
2. Rodent models of the BBB under dynamic conditions recapitulate rodent brain permeation data (Figure 6).
3. Dynamic models are a better approximation of *in vivo* conditions compared to static models, which in general are not very useful predictors of drug passage across the BBB *in vivo*. This is in particular true when comparing the static vs. dynamic models side by side (20).
4. Use of endothelial cells derived from diseased brain and grown under dynamic conditions are a close representation of data *in vivo*. This was demonstrated in the case of temporal lobe epilepsy (49–52, 58, 59, 61, 95, 96). We were able to directly compare blood and brain levels *in vivo* (before temporal lobectomy) to luminal and abluminal levels in a DIV-BBBB model that was prepared by using endothelial and glial cells taken from the resected tissue. In particular, we reported that *in vivo* and *in vitro*, the anti-epileptic drug carbamazepine was metabolized by endothelial P450 enzymes into a neurotoxic, seizure-promoting agent [quinolinic acid (50)].
5. A personalized medicine approach where cells from the human pathology to be studied are directly grown *in vitro* is the most promising approach to modeling of pharmacokinetic properties of therapeutics. This is possible in diseases where resected tissue is available, such as multiple drug-resistant seizure disorders or brain tumors such as glioma, oligodendrogliomas, etc.

#### Organ-on-a-chip and Microfluidics Applied to the BBB (BBB-on-a-chip)

Groundbreaking work regarding OOAC has been addressed in recent literature. BBB-on-a-chip is an *in vitro* BBB model as an OOAC setting allowing for further physiological study beyond the limitations of the conventional Transwell model (8–11). Griep and colleagues reported a newly designed BBB-on-a-chip model coated with rat collagen-1 in a microfluidic platform (two compartments). The model consisted of a two-layer membrane-based device composed of poly-dimethylsiloxane (PDMS) with a top and bottom channel with the Transwell membrane placed in the middle portion of the chip. A monolayer of immortalized human brain endothelial cells (hCMEC/D3) was seeded in the top channel. Compared to the Transwell model, this BBB-on-a-chip schematic achieved higher static and dynamic TEER values, the latter of which involved the addition of shear stress (5.8 × 10^−1^ Pa) and assessed by TNF-α treatment (8). This suggests that this BBB-on-a-chip model not only yields a tighter monolayer, but also shows that the addition of shear stress can better approximate the tightness of BBB endothelial layer. This is similar to what observed with the DIV-BBB. The model can be enhanced by co-culture with astrocytes and pericytes, as it is shown that the crosstalk between these cell types (as components of the neurovascular unit) not only fundamentally affects the tightness of the epithelium but also exerts cellular/molecular regulation over brain endothelial cells.

When developing *in vitro* models of the BBB, it is also important to consider the imaging techniques needed for analysis, which, in turn, will determine the suitability of a BBB-on-a-chip model vs. another. The BBB-on-a-chip model designed by Salman and colleagues in 2020 addresses this point. Their model involves human brain microvascular endothelial cell (TY10) monolayers seeded on a 3D microfluidics PDMS-chipset with the purpose of developing microvessel structures and geometry (9). Chips were also grafted on extracellular matrix (ECM) substrates (modified collagen matrix) to afford physiological rigidity. The study aimed to assess if this model was able to be imaged by fluorescence and electron microscopy, which indeed were able to achieve. The ability for these models to be imaged is crucial for drawing conclusions and establishing future directions of study. A limitation to this model, however, is the inability of determining BBB integrity by the standard electrode-based TEER. Despite this, fluorescence imagery of tagged monoclonal antibodies served as a proxy measure.

Neuroinflammation is a serious complication (or possible causation) of neurological illness, and arising evidence puts the BBB at its core. Inflammation in the CNS can be due to peripheral immune activation or driven by microglia and other resident cells. These processes accentuate the leakiness of the BBB, allowing the peripheral immune system to induce neurodegeneration. Multiple disease like MS, AD, and NeuroAIDS have some level of neuroimmunological insult as part of their hallmark pathologies. Having models that can bring about deeper insight into the intricate interplay between the BBB and the immune system is of vital value. Herland and colleagues in 2016 devised a BBB-on-a-chip model of neuroinflammation (10). Their model consisted of microfluidic channels with hollow cylindrical collagen gels cultured with primary human brain endothelial cells (inner surface) and astrocytes and pericytes (outer surface microvessel). Upon insult to the chip model using TNFα, the group observed a similar response to BBB disruption (due to elevated secretion of G-CSF, IL-6, and IL-8) as in cases of stroke, findings that were not mimicked in the Transwell model. This is further evidence affirming the promising results that can be obtained with OOAC *in vitro* BBB models incorporating the geometrical properties by which the BBB finds itself in while considering the inflammatory nature of neurological pathology. Metabolism is especially important toward BBB vitality and integrity. Energy expenditure and transfer across the neurovascular unit is hard to model using current Transwell methods and such models do not accurately reflect metabolic phenotype observed *in vivo* (20). Maoz et al. (11) addressed this by developing three coupled BBB chips in co-culture with primary human brain endothelial cells, pericytes, astrocytes, and neurons. The group also assembled uncoupled chips for experimental comparison of the models. Following studies involving human-on-a-chip settings, this project found that the metabolic fluidity and transport between compartments of the chip representing the brain and the periphery was conserved in resemblance to findings *in vivo* as evaluated by glucose, glutamate, GABA, and methamphetamine synthesis and transport. In addition, results in the coupled co-culture system better mimicked *in vivo* manifestations as compared to static, monoculture, and uncoupled chips. Considering coupled chipsets in representations of different brain compartments is yet another consideration for making OOAC *in vitro* models of the BBB.

Curiously, the addition of methamphetamine transport in the previous study could also be extrapolated to the study of drug delivery and nanomedicine as nanoparticle functionalization is vital for the transport of drugs across the BBB. Ahn et al. (97) in 2018 engineered a microfluidic chip model involving a 3D astrocytic network in co-culture with pericytes and human brain microvascular endothelial cells to investigate the mechanics of nanoparticle transport across the BBB. Compared to 2D human astrocyte cultures, the 3D network of human astrocytes presented downregulated gliosis (determined by vimentin and LCN2 analysis) and improved AQP4 polarization. The study synthesized an HDL-mimicking nanoparticle [with apolipoprotein 1 (A1)] for testing the model's ability to imitate selective BBB transcellular transport. Results showed that the nanoparticle ensemble utilized scavenger receptor class B type 1 (SR-B1)-mediated transcytosis as seen in prior *in vivo* projects from this same research group. However, each nanoparticle ensemble comes with its own sets and challenges, which may or may not be applicable to this model.

#### Brain Organoids and the Importance of the BBB

The brain is a complex organ that spans from the physical manifestations of disease to the psychological aspects of being human. The complexity of its extensive synaptic network continues to challenge ongoing research as many neurological illnesses remain with ill-defined etiology despite major advances in past years. The field of *in vitro* modeling of the brain has highly considered the development of “human-on-a-chip” formfactors to decrease the extent of animal work done for research as it is known to be of elevated cost, training, and longevity aside from the ethical and legal considerations associated with *in vivo* studies, not to mention that results from animal work may not translate accurately to human physiology. Because of this, the development of organoids (which is a technology that has been used for decades) derived from human stem cells is becoming more and more accessible to the bench and improving its resemblance to findings *in vivo*. However, modeling the brain *in vitro* carries a long set of challenges that must be overcome, one of which is to properly establish an *in vitro* BBB that fulfills the function of a tight and selective histological barrier between brain extracellular fluid (BECF) and the peripheral vasculature.

Despite the BBB not being an organ, it is still of much relevance to brain organoid research. Due to the barrier having an intricate orchestration between the periphery and the BECF, it is important to assess if the synthesized brain organoid develops a functional BBB and vascular-like structures. Achieving this would warrant findings being more realistic and can provide better insight as to how the whole brain (or certain regions) can become affected due to BBB disruption. In 2019, Cakir et al. (98) expanded on the BBB-like characteristics of their model. Their human cortical organoid (hCO) setup considered the lack of microvasculature structures, which indeed would misrepresent the nature of nutrient exchange in the developing brain. However, differentiation of human embryonic stem cells (hESCs) into endothelial cells required ectopic expression of human ETS variant 2 (ETV2). In short, expression of this transcription factor yielded BBB-like functions to their experimental hCO models due to expression of α-ZO1 in addition to the presence of differentiated astrocytes and pericytes (confirmed by fluorescence staining), and with TEER values comparable to other 3D models in the literature. The group also tested the biological responses of the mimicking microvasculature to amyloid β_1−42_ (known biomarker for AD, view in section*Translation to Human Disease*) and found that certain variants (oligo vs. fibril) were able to disrupt the tightness of the BBB as expected. This serves to show that brain organoid research can truly benefit from incorporating BBB-like capabilities as these models can also be used for further research of neurodegenerative disease. More discussion about brain organoids for clinical and neuropathological research can be found here (99, 100).

Brain organoids can also be of excellent use for determining drug penetration into the CNS. Bergmann et al. introduced a protocol for “BBB organoids” to address this issue. The protocol indicates the formation of a tri-culture spheroid using brain endothelial cells, astrocytes, and pericytes with differing incubation times (incubation times may vary based on the drug under study) (101). Protocol goes more in-depth with respect to stepwise wet-lab procedures; however, it is of much interest to see that methodologies are arising for testing compounds to cross the BBB in an *in vitro* setting as opposed to *in vivo*.

### Translation to Human Disease: Current BBB *in vitro* Models of Neurological Disease

The etiology of many neurodegenerative diseases continues to be ill-defined. Despite major investigational strides in recent years, many pharmacological and/or biologic options on the market can either solely alleviate symptoms and/or slow down disease progression. More investigation is needed to further elucidate the course of illness and better define the stages of disease in a more quantitative rather than qualitative form. Greater insight into diseases like AD, Parkinson's disease (PD), MS, and seizures, among others, could be obtained by analyzing the pathology of the BBB. There has been new evidence suggesting BBB involvement in the pathological course of illness for AD, MS, and seizures (102–105). In this section, we explored the literature regarding the employment of *in vitro* BBB models to further develop therapies and diagnostics and contribute knowledge to the pathophysiology of a few abovementioned conditions. This is by no means an exhaustive representation of all the research up to the present; however, it recovers main points of interest in research (and potential modifications for extended investigation) that could make their way into the practice of medicine. In addition, we will also cover the neurological implications of infectious agents (mainly viral in nature) that pose major concern to public health due to their extensive prevalence, incidence, and infectivity.

#### Viral Infection of the Brain

It is widely known that a plethora of infectious diseases, including sexually transmitted infections (STIs) and parasitic infections, have the ability to infect the CNS and cause illness. This can lead to a series of neurological deficits that can be both focal and generalized, and of which may or may not be diagnosable via electroencephalography (EEG), serology, and/or CSF testing. In this section, we discuss some infectious disease agents of high relevance to public health due to their widespread range, high prevalence, and infectivity. In addition, some of these illnesses are yet to be fully curable by pharmacological and/or biological therapies, and vaccine candidates are currently being assessed or yet to be developed. Further understanding of how the BBB comes into play regarding these infections can provide new avenues toward developing targeted therapy, improving drug delivery mechanisms, and contribute knowledge about the CNS pathophysiology brought by these pathogenic agents that could also make their way to innovate medical care.

NeuroAIDS is an end-stage process of HIV infection (106–108). *In vitro* models of the BBB have been used to determine whether endothelial cells from the brain can act as reservoir of HIV or as site of viral entry into the brain (55–57, 108–110). A number of different devices were used, spanning from *in vitro* 2D cultures to the DIV-BBB. A significant finding was that simian immunodeficiency virus (SIV) could persist in endothelial cells cultured under flow conditions for months and replicate/mutate *in vitro* (56). In addition, different models were used to test therapeutic efficacy of AIDS treatments (111–114).

Lessons learned from HIV/SIV studies on the BBB are currently used to study mechanisms and treatments of Severe Acute Respiratory Syndrome Coronavirus 2 (SARS-CoV-2), the causative agent of COVID-19. This novel coronavirus outbreak has fueled serious mitigation efforts worldwide since December 2019. Given multiple reports of COVID-19 patients developing neurological disease (115–118), there is reason to believe that this viral infection may span more than just respiratory illness. For the purposes of this review, we will not go over the intricacies of the SARS-CoV-2 infection cycle, but rather investigate the literature regarding the involvement of the BBB during the course of illness and how relevant are BBB *in vitro* models toward further understanding the neurological effects of COVID-19. Multiple case studies have reported neurological complications to COVID-19 (115–118). Some of these have reported no presence of SARS-CoV-2 genetic material in CSF via polymerase chain reaction (PCR) testing (115, 116) whereas others have documented SARS-CoV-2-positive CSF samples upon PCR (118), and a few mentioning that BBB disruption may be possible (117). Given how recently this virus has emerged, exact mechanisms as to how SARS-CoV-2 is causing neurological disease remains to be fully defined. The BBB can become affected during COVID-19 course of illness. Given the cytokine storm being the hallmark pathology of COVID-19, there is much concern as to how the BBB can be disrupted. The main cytokines released during the process of heightened inflammation include interluekin-6 (IL-6), interluekin-1β (IL-1β), tumor necrosis factor-α (TNFα), chemokine C-C motif ligand 2 (CCL2), and granulocyte-colony stimulating factor (GM-CSF) (119). These pro-inflammatory cytokines direct systemic inflammation against SARS-CoV-2 which is thought to be initiated by innate immune mast cells. Literature shows that SARS-CoV-2 can achieve cellular entry via angiotensin-converting enzyme 2 (ACE2) receptors, which are highly expressed throughout many tissues, including neural tissue (i.e., neurons and glia). It is thought that SARS-CoV-2 can access the brain by either infecting the olfactory epithelium retrogradely transporting to the brain via the olfactory bulb and/or by infecting endothelial cells of the neurovascular unit (118, 119). The latter of which allows loss of BBB integrity and internalization of the virus to the CNS, further infecting neurons and glia.

A recent *in vitro* model of COVID-19 and the BBB has shown that the spike protein S1 promotes loss of barrier integrity in an advanced 3D microfluidic model of the human BBB. Evidence provided also suggests that the SARS-CoV-2 spike proteins trigger a proinflammatory response on brain endothelial cells that may contribute to an altered state of BBB function (120). A sophisticated model of the BBB was used, which allowed us to expose endothelial cells to parenchymal factors (Matrigel) that promote tube formation and luminal flow. A one-way, non-recirculating flow system was used to perfuse the lumen. Endothelial barrier formation was monitored by TEER and FICT dextran permeability. Both were affected by spike protein 1 h after application. TEER monitoring used relatively high frequencies (400–48,000 Hz), which may be optimal for the geometry of the microfluidic device employed but are in the high range of commonly used frequencies. A study on human patients has shown that the results cited above may be true in a clinical setting (121): patients with advanced COVID-19 illness presented with high levels of serum S100B, a marker of BBB integrity (122, 123). Thus, combining the use of BBB *in vitro* models with S100B as a marker of BBB disruption could yield more definitive conclusions with regard to how COVID-19 is causing neurological diseases of the brain.

Not only is S100B an important marker to consider, but it is also of interest to further understand how the spike protein of SARS-CoV-2 could potentially trigger BBB disruption and which domains exert such activity. In June 2020, Buzhdygan et al. (124), using a 3D microfluidic hydrogel *in vitro* model seeded with a monolayer of human brain microvascular endothelial cells (hBMVECs), were able to show that SARS-CoV-2 spike protein subunit 1 (S1) is capable of causing BBB disruption during COVID-19 illness. Although mechanisms are still being elucidated, the research group found that S1 induced activation of hBMVECs, resulting in overexpression of adhesion molecules, recruitment of pro-inflammatory cytokines, and increased expression of matric metalloproteinases. In summary, all of these indicators suggest that S1 induces loss of BBB tightness, which can result in neurological insult in COVID-19 patients. Albeit groundbreaking, utilization of other *in vitro* models such as OOAC and brain organoids could also be considered to extend our knowledge of how COVID-19 can lead to cortical damage. Notwithstanding, queries remain regarding monoculture studies (even those including shear stress conditions in microfluidic settings) about the role of astrocytes and pericytes with respect to, in this case, viral infection. Further study may also consider which key cytokines could be targeted to control or prevent viral infiltration to the brain or further neuroinflammation from occurring.

#### Alzheimer's Disease

AD is the most common type of dementia. It is also the sixth leading cause of death in the United States with cases estimated to reach 16 million by the year 2050 (Alzheimer's Association). For the past decades, major insight into its etiology and pathology has made way for the development of pharmaceuticals that can aid in alleviating symptoms; however, there are no current therapies that can slow disease progression. The pathological hallmarks of AD are the accumulation of amyloid-β (Aβ) plaques and neurofibrillary tau tangles, the latter of which being further subclassified into total tau (T-tau) and phosphorylated tau (p-tau), indicating extent and rate of disease advancement, respectively (125). Accumulation and propagation of these proteins over time (especially in the hippocampal region) lead to progressive cognitive decline and memory loss that can make patients completely reliant on caregivers. Current hypotheses mainly focus on the prion-like fashion of AD pathogenesis with some starting to look into the neuroinflammatory influences of the disease and BBB integrity involvement (102, 126). Discussion regarding the pathogenesis and pathophysiology of AD has been extensively described elsewhere (125), for this section will focus on the feasibility of BBB *in vitro* models to be used to further investigate neurodegenerative diseases of extensive pathological development like AD.

Cerebrovascular disease has an intricate relationship with AD pathogenesis. Risk factors such as hypertension, hyperlipidemia, and diabetes are common to many cardiovascular diseases that in turn increase the risk for developing cerebrovascular events like stroke. Some clinical studies have demonstrated that AD patients may have cardiovascular comorbidities that predated their dementia diagnosis, making them more prone to develop cerebrovascular pathology that is known to compromise BBB integrity (126, 127). The neurovascular unit acts as a highly responsive syncytium to vascular triggers, whereby endothelial cells can regulate gene expression of tight junction proteins to maintain TEER at optimal levels dependent on sheer stress and hemodynamic factors (31). Conditions of vascular disease in the brain can exacerbate BBB disruption, which could ultimately lead to either initiation or prolongation of Aβ and tau protein deposition in brain extracellular fluid (BECF) (126). Biomarkers of vascular insult predate changes in CSF biomarkers of AD pathogenesis (Aβ42, T-tau, and p-tau) and symptoms of cognitive decline (128). Repercussions of vasculopathy that ultimately trigger BBB disruption are truncated glucose transport, loss of tight epithelium, pericyte degeneration, leakage of peripheral fluid into the CNS, induction of neuroinflammation, and a decrease in cerebral blood perfusion (CBF) (126). It is important to gain from this discussion that vascular insult should ultimately equate BBB disruption, therefore making it quite clear that understanding the consequences of BBB disruption due to vasculopathy could add more insight into AD pathogenesis. Modeling AD-associated vasculopathy settings *in vitro* will be the best way to determine the pathophysiological mechanisms while identifying the key agents at play. In addition, given the relevance of ApoE4 as a risk gene for AD, understanding how this protein interplays with the BBB is yet another possible scenario to put under scrutiny. All of this discussion serves to show how important understanding cerebral vasculopathies can be toward understanding AD and how such can make the BBB more permeable to the periphery. Despite all this, it is reasonable to question if such findings can be replicated at the *in vitro* level. Further discussion comparing Transwell and dynamic *in vitro* BBB models can be found elsewhere (20). However, the main question remains, can a viable BBB *in vitro* model for AD be developed given the stark difference in time scale for disease pathogenesis and model development?

It is widely known that one of the pathological hallmarks of AD is the accumulation and propagation of Aβ plaques. However, much attention has been drawn to further characterize biomarkers of disease such as Aβ42. Research has shown that Aβ42 has a higher capacity to promote aggregation when compared to Aβ40 (125). A low Aβ42/Aβ40 ratio has been observed to drive AD pathology as Aβ42 seeding promotes nucleation and further deposition of plaques. Currently, a few studies have explored pharmacological measures for both diagnostics and drug delivery utilizing BBB *in vitro* models. A study evaluated the possibility of D-amino acid peptides to be used as a diagnostic tool (129). D-amino acid peptide-1 (D1) is a highly lipophilic peptide that has the ability to bind to Aβ42. Given that the BBB allows for lipophilic content to cross the barrier with ease, it can be reasonably thought that this type of molecule can cross the barrier and reach Aβ42 plaques. Using a Transwell *in vitro* BBB model composed of a co-culture of rat brain microvascular endothelial cells (RBMECs) and rat astrocytes, the study showed that D1 can permeate the BBB in a dose-dependent manner despite finding a propensity toward D1 brain efflux rather than influx. Following reports of increased astrocytic P-glycoprotein expression in astrocyte-endothelial cell co-cultures (130), it was reasonable to prove whether astrocytic influence on endothelial cells contributed to D1 efflux. Effectively, D1 influx was ameliorated in the presence of verapamil, a P-glycoprotein inhibitor (129). Further research is warranted, however. Given the dynamic features of the BBB and the hemodynamic factors that contribute to its dynamic nature, it is important to compare these results with dynamic *in vitro* models of the BBB. Earlier studies have explored the roles of dynamic activity and shear stress in *in vitro* BBB models at length (20, 31). Further research involving the dynamic features of the BBB could better characterize the fitness and accuracy of potential diagnostic molecules.

Understanding how the BBB is compromised during AD will pave the way forward for more directed diagnostics and potential treatments insofar as the pathophysiology of such disruption, contributing to AD pathology itself, is elucidated. Shin and colleagues developed a novel 3D monolayer microfluidic model, composed of human brain endothelial cells (bECs), to investigate the increased permeability of the BBB during AD (131). The model is grouped in what the group calls “microchannels” filled with hydrogels as a fluid communicative medium. The model was also co-cultured with neural progenitor cells (NPCs) expressing familial AD (FAD) mutations in APP and APP/PSEN1 genes, known genes to control Aβ nucleation. Not only was their model able to mimic increased BBB permeability conditions as in AD, but findings also revealed reduced expression of adherence molecules (i.e., Claudin-1, Claudin-5, and VE-cadherin) in bECs, increased levels of reactive oxygen species (ROS), metalloproteinases, and IFNγ. Moreover, the model indicates that reduction of Aβ fibrils leads to decreased BBB permeability. To analyze the extent of neural damage brought by BBB disruption, the group introduced a neurotoxic agent (thrombin). Furthermore, evaluation of the BBB protective effects of etodolac and beclomethasone was also assayed. Conclusively, the study found that neurotoxin (thrombin)-induced cell death upon BBB disruption caused by Aβ fibril deposition and etodolac (but not beclomethasone) showed to reverse the effects of BBB disintegration by increasing the expression of cell–cell adhesion molecules. This is the start of promising research in the field of neurodegeneration despite this study utilizing a monolayer to simulate the BBB.

Other studies have utilized *in vitro* BBB models to study the suitability of potential drug delivery molecules for treating AD, including those coupled with nanoparticles. Past research brings evidence suggesting that non-steroidal anti-inflammatory drugs (NSAIDs) may be useful in decreasing the synthesis of Aβ42 proteins by modulating γ-secretase activity (132, 133). A group entertained the idea of utilizing nanoparticles for drug delivery of NSAIDs as a possible treatment for AD (134). Previous work from this research group determined that the NSAID, flurbiprophen, can inhibit the synthesis of Aβ42 in Aβ42-producing Chinese hamster ovary (CHO) cells (133). Utilizing a co-culture Transwell model employing APP751 overproducing CHO cells and mouse brain endothelial cells (bEnd.3), it was determined that flurbiprophen-embedded polylactide (PLA) nanoparticles did decrease the production of Aβ42 in a dose-dependent manner. The authors suggested further study of nanoparticle functionalization with apolipoproteins, given that apolipoproteins (more specifically ApoA4 and ApoE) may aid drug transport across the BBB (135–137). This serves to show that *in vitro* BBB models can also be used to further investigate the role of apolipoproteins not only for determining facilitation of drug delivery but also to the explain their key role in AD pathogenesis with major emphasis on the known risk gene ApoE ε4 allele.

This can lead to question what roles do ApoE proteins play pertaining to BBB integrity. New evidence suggests that ApoE regulates the tightness of BBB (138). It has been shown that the astrocytic ApoE ε4 isoform poses the major genetic risk toward developing AD, and recent evidence has shown that lack of ApoE disrupts the BBB (102, 125, 126). A study (138) employed the use of a triculture Transwell BBB *in vitro* model composed of primary culture mouse brain endothelial cells, pericytes, and ApoE3/4-*knock-in* astrocytes (138, 139). ApoE3-*knock-in* BBB model cultures yielded significantly tighter BBB compared to ApoE4-*knock-in* BBB model cultures (compared to ApoE-KO and wild-type culture controls), suggesting that ApoE-driven BBB permeability may be isoform-dependent. Given evidence that endothelial PKCη phosphorylation of tight junction proteins (i.e., occluding, claudins, cadherins, etc.) promotes BBB integrity, it was reasonable to evaluate whether astrocytic ApoE modulated tight junction phosphorylation in endothelial cells in an isoform-dependent manner (102, 138, 140). Astrocytic-ApoE3-*knock-in* BBB models showed similar BBB phenotype as wild-type BBB models in comparison to astrocytic-ApoE4-*knock-in* BBB models that demonstrated reduced PKCη phosphorylation of tight junctions and overall PKCη concentration in lysate. This suggested that the ApoE4 isoform, in accordance with the literature, may play a central role in BBB permeability that could contribute to AD pathology. More insight could be gained by comparing this Transwell model with other dynamic *in vitro* BBB models incorporating the use of primary triculture cells to better resemble the true environment of the BBB.

#### Multiple Sclerosis

MS is an autoimmune neurodegenerative disease of unknown definitive cause. The hallmark of the illness is the progressive or relapsing attacks on myelin sheaths (demyelination) orchestrated by peripheral immune infiltration into the CNS. The frequency and extent of demyelination (and subsequent manifestation of symptoms) allows the characterization of relapsing–remitting MS (RRMS), primary progressive MS (PPMS), and secondary progressive MS (SPMS) (Multiple Sclerosis Society). Differential diagnostic criteria require clinical and imaging examination proving the presence of present or past inflammation [dissemination in time (DIT)] and spatial extent of inflammation [dissemination in space (DIS)] per the McDonald Criteria for the Diagnosis of Multiple Sclerosis [revised in 2017 (141)]. Optic neuritis is one of the most common initial manifestations of MS (142). Due to infiltration of peripheral immunity, there is no doubt that BBB integrity is lost, thus compromising the immune privilege of the CNS and triggering local neuroinflammation that can exacerbate pathology.

The use of *in vitro* BBB models can shed light onto devising the key players, mechanisms, and determining the initial triggers for immune infiltration to occur. Currently, therapeutic interventions include the use of oral steroids and intravenous infusions, the latter of which is becoming more common. Targeted therapy that (1) effectively dampens pro-inflammatory signaling and (2) ameliorates BBB restoration would be better suited to attempt slowing disease progression and/or reducing the occurrence of relapses. However, to develop these interventions, it is important to first elucidate the intricacies of cellular signaling, mechanisms, and triggers that govern the crosstalk between the BBB and the peripheral immune system. On the one hand, cytokines like TNFα, IL-6, and IFNγ are known to increase BBB permeability (aside from the action of metalloproteinases), whereby the tightness of the endothelium is disrupted by attacking cellular adhesion and tight junction proteins (105). On the other hand, neurotropic viruses such as Epstein–Barr virus (EBV) have been detected in multiple MS patients and are considered risk factors for the disease as these could initiate pathogenesis (143). Many of these findings have been done *in vivo* using the model of experimental autoimmune encephalitis (EAE); however, there is lacking literature involving *in vitro* BBB models that further elucidate the mechanisms of BBB disruption during MS immunopathology.

IFNβ is an anti-inflammatory cytokine with the ability to promote BBB integrity and an effective treatment for patients with RRMS (144). A report employing the use of a Transwell co-culture composed of human brain microvascular endothelial cells (HBMECs) and rat astrocytes evaluated the stabilizing effect of sera from MS patients treated with IFNβ-1b, suggesting the presence of an unknown factor that is promoting BBB disruption (145). BBB permeability was assayed following previous methodologies testing the permeability of inulin and sucrose and TEER. The study found that both sucrose and inulin permeability in Transwell models treated with sample sera from IFNβ-1b-treated MS patients was significantly decreased compared to Transwell models treated with sera from IFNβ-1b-untreated MS patients. MRI scans from the study participants that donated sera were taken using gadolinium contrast and sorted by treatment with IFNβ-1b to correlate with *in vitro* BBB Transwell models' inulin permeability values. Subjects under IFNβ-1b treatment with low T2 lesion loads (0–5) showed significantly lower BBB permeability to inulin with no significant differences upon mid (6–15) T2 lesion loads. This confirms their findings *in vitro* that IFNβ-1b treatment does seem to restore BBB integrity but to a certain quantifiable extent, leading to question the mechanism of IFNβ-1b-mediated control and the agent(s) partaking in the process.

Neurotropic virus infections have been suggested to induce MS pathogenesis. EBV has been detected on multiple MS patients leading researchers to study this and other viral vectors (e.g., Herpesvirus 6) that could potentially initiate and/or worsen MS pathology; however, attention has also been drawn to endogenous viruses like MS-associated retrovirus (MSRV). It is a member of the human endogenous retrovirus W (HERV-W) family that infected the germline and has been passed down through generations as a consequence of human evolution from primate ancestors (146, 147) with the ability to induce inflammation via Toll-like receptor 4 (TLR4). This virus has also been isolated from the CSF of MS patients in the past (148), indicating that activation of gene transcription of this endogenous viral genome could be implicated in MS pathogenesis due to viral proteins triggering inflammation.

A research group evaluated this query utilizing a monolayer *in vitro* BBB model consisting of immortalized human brain endothelial cells (HCMEC/D3) to evaluate mechanisms of inflammation mediation in response to recombinant MSRV envelope protein (Env-ms) (149). Data revealed that Env-ms triggers significant ICAM-1 overexpression in HCMEC/D3 monolayers and induces brain endothelial-cell release of IL-8 and IL-6 in a concentration-dependent manner when compared to TNFα-treated cultures. In addition, due to previous reports of TLR4 being expressed in HCMEC/D3 cell lines, the study assessed the involvement of TLR4-mediated inflammation in response to Env-ms via siRNA directed against TLR4. Indeed, a drastic decrease in ICAM-1 expression was found (compared to non-transfected and control siRNA-transfected cultures) in TLR4 siRNA + Env-ms samples. This clearly illustrates that TLR4-directed inflammation in the presence of MSRV envelope protein increases BBB permeability. Moreover, the study found that HL-60 cell transmigration through the *in vitro* BBB model was enhanced in the presence of Env-ms, further indicating that MSRV increases immune cell migration *in vitro*, which may well trigger neuroinflammation. Additional studies could expand upon this model by including co-culture experiments, with astrocytes and pericytes being the key members of the BBB. Insight from these studies could elucidate further methods of regulation that may reinforce or disprove some of the abovementioned findings or, preferably, uncover other yet to be defined modes of interplay throughout the neurovascular unit.

One of the prime pathological factors where further definition is needed is the transmigratory process of immune cells across the BBB. A recent study sought to clarify the effects of disease utilizing an *in vitro* BBB Transwell model of human brain endothelial cells (HBECs) (cultured in presence of TNFα and IFNγ) to test the mechanisms of immune cell transmigration and its limitation in the presence of fingolimod (an S1P inhibitor) (150). S1P is a G-protein-coupled receptor modulator whose signaling has strong repercussions on BBB integrity due to its action on S1P receptors (S1PR). S1PRs are overexpressed in resting T- and B-cells and fingolimod can block their exit from the thymus and lymph nodes by directly inhibiting S1PR activity (150). Their Transwell co-culture experiments with peripheral blood mononuclear cells (PBMCs) found that fingolimod (comparing Transwells that were co-cultured with PBMCs extracted from healthy, MS participants treated, and untreated with fingolimod) was able to alter subpopulations of T-cells, B-cells, and natural killer (NK) cells. The research group's extensive cell-type-specific dataset suggests that, albeit statistically insignificant, CD4^+^ T-cells tended to have a higher migratory capacity compared to CD8^+^ T-cells from fingolimod-untreated MS patients; however, significant reduction in T-cell migration in the presence of fingolimod was observed. In addition, fingolimod increased the proportions of CD8 and CD56 dim-expressing T- and NK cells that correlate to MRI studies of MS mentioned elsewhere (151).

This is yet another example of how *in vitro* BBB models can provide further insight into the cellular mechanisms governing immune cell migration in an in-depth fashion that may be more difficult to achieve at the *in vivo* level. Ongoing studies should also consider the influential role of astrocytes on the regulation of endothelial cell expression of cellular adhesion and tight junction molecules, and by consequence, influencing the resistance/integrity of the BBB *in vitro* model that ultimately results in altering transmigration of immune cells.

## Conclusions and Future Directions

Given the transformation of *in vitro* models throughout the last decades, it is easy to predict that exciting changes are ahead. There are a few “hot topics” that we did not summarize that need to be mentioned. One relates to the materials used as substrates for cellular growth. Graphene is an allotrope of carbon consisting of a single layer of atoms arranged in a two-dimensional honeycomb lattice. A review of its properties and history details its use in nanomedicine (152). For DIV-BBB cell culture, hollow fibers were spiked with graphene to show increased transmural pore size and increased electrical conductivity (153). These may be beneficial because increased pore size will improve passage of nutrients across the fiber wall, while increasing conductivity should decrease the elevated hollow fiber background resistance when measuring TEER. In addition, the authors show improved cell adhesion without adding collagen or fibronectin as adhesion molecules. Finally, the larger whole sizes allowed for enhanced contact-forming glial end-feet from the abluminal to the luminal side.

Extracellular microvesicles and exosomes have received a great deal of attention in the field of BBB research as means of tissue signaling (154, 155), markers of disease (156), or therapeutic carriers (157). One of the main issues relates whether passage of microvesicles/exosomes occurs at the normal BBB. In our opinion, there are no definitive answers to this important question. There is, however, evidence that tumor-derived extracellular vesicles can breach the BBB and that vesicles cross the BBB by transcytosis (158). BBB research has and will rely on *in vitro* models. These are of increasing sophistication and may be ready for use in a human-on-a-chip technology. Several issues remain unsolved, including the exact vascular counterpart that is being modeled [venous, arterial, or capillary, see (30)] and the exact amount of shear stress required to mimic the *in vivo* vessels. The use of patient-derived cells may improve the predictive value of these models for drug delivery. Furthermore, per our extensive discussion regarding modeling of disease, there are limitations to brain endothelial cell monolayer models as these lack the communicative cross-regulation between astrocytes and pericytes. Models that employ the use of co-culture models obtain data that more closely resemble the BBB, therefore making monoculture experimentation merely a starting point toward properly defining BBB integrity maintenance. Lastly, complementing *in vitro* BBB modeling with known genetic markers of disease may provide groundbreaking insight into BBB pathobiology and also bridge/confirm findings done *in vivo* with the added feature of elucidating cellular and molecular mechanisms and key players.

## Author Contributions

DJ and AW-M wrote the manuscript. DJ and MD compiled data to produce the figures. All authors contributed to the article and approved the submitted version.

## Conflict of Interest

DJ is the Scientific Director of Flocel, Inc., a company manufacturing the DIV-BBB system described in this review. AW-M participates in a graduate unpaid internship at Flocel, Inc. under the mentorship of DJ. The remaining author declares that the research was conducted in the absence of any commercial or financial relationships that could be construed as a potential conflict of interest.
